# Analysis of the effect of ALA-PDT on macrophages in footpad model of mice infected with *Fonsecaea monophora* based on single-cell sequencing

**DOI:** 10.1515/med-2024-1132

**Published:** 2025-01-13

**Authors:** Wenyi Chen, Xuelin Wu, Muhammad Danish Yaqoob, Kangxing Liu, Yanqing Hu, Xiuling Ke, Yongxuan Hu

**Affiliations:** Department of Dermatology and Venereology, The Third Affiliated Hospital, Southern Medical University, Guangzhou, China; Guangdong Provincial Key Laboratory of Bone and Joint Degeneration Diseases, Guangzhou, China; Department of Dermatology and Venereology, The Third Affiliated Hospital, Southern Medical University, 183 West Zhongshan Road, Guangzhou, China

**Keywords:** photodynamic therapy, chromoblastomycosis, *Fonsecaea monophora*, macrophage, single-cell sequencing

## Abstract

Chromoblastomycosis (CBM) is a chronic neglected fungal disease that causes serious damage to the physical and mental health of patients. 5-Aminolevulinic acid photodynamic therapy (ALA-PDT) has garnered significant attention in the recent era for the treatment of CBM and has exhibited promising effects in several clinical case reports. We established a mice footpad infection model with *Fonsecaea monophora* and analyzed the impact of PDT treatment on the immune response of macrophages using single-cell sequencing. The results showed that infection of the mouse footpad skin with *F. monophora* results in an increase in inflammatory cells, primarily mononuclear-macrophages, with the activation of complement and enhancement of cell chemotaxis, leading to upregulation of anti-infection-related pathways. After ALA-PDT treatment, the number of inflammatory cells decreased, while macrophages upregulated the expression of antigen-recognition-related genes, enhancing phagocytosis and autophagy-related biological functions.

## 1 Introduction

Chromoblastomycosis (CBM) is a chronic, granulomatous, and suppurative skin and subcutaneous tissue infection, which is attributed to the presence of dematiaceous fungi. The genus *Fonsecaea spp.* serves as the most prevalent etiologic agent in Asia, with *Fonsecaea monophora* being among the most frequently encountered agents in the southern regions of Mainland China, as reported in prior studies [1,2,3]. Regardless of the causative agent, standardized therapeutic approaches for infections arising from *Fonsecaea* spp. remain unavailable. The recommended therapies include surgical resection, local or systemic antifungal therapy, cryotherapy, hyperthermia, and laser therapy. First-line therapy is recommended with systemic antifungal treatment, with Itraconazole and Terbinafine being commonly used antifungal agents. However, the long-term oral use of antifungal drugs can cause significant hepatotoxicity and nephrotoxicity, which reduces patient compliance, and the increasing fungal resistance makes treatment more difficult. In addition to primary small skin lesions that can be effectively treated with surgical resection, most moderate to severe CBM patients are difficult to cure due to their stubborn condition.

In recent years, photodynamic therapy (PDT) has shown good results in the treatment of CBM, and it has been reported that PDT alone or combined with antifungal drugs can significantly improve skin lesions [4,5]. 5-Aminolevulinic acid (ALA) is one of the most commonly used PDT photosensitizers in clinical practice, and Aminolevulinic acid photodynamic therapy (ALA-PDT) has gradually demonstrated its unique advantages as a clinical adjuvant treatment for CBM. However, the specific mechanism of its action in the treatment of CBM remains unclear and needs to be experimentally explored to provide more comprehensive scientific evidence for clinical practice.

As a crucial component of the immune system, macrophages play a vital role in resisting pathogen invasion and regulating inflammatory responses. *In vivo*, the inflammatory process is subject to very strict regulation, as an imbalance between pro-inflammatory and anti-inflammatory cytokines can lead to damage to the cells and tissues. Macrophages play a key role in this process by regulating the initiation and resolution of inflammation through three main functions: phagocytosis, antigen presentation, and immune regulation [6,7,8]. Macrophages play an important role in regulating fungal growth, and it has been reported in the literature that intracellular vacuoles in the cytoplasm of skin macrophages contain CBM pathogenic fungi cells, indicating that activated macrophages have inhibitory and immune-killing effects on fungi [9]. Moreover, Langerhans cells isolated from mouse skin have phagocytic activity toward pathogenic fungal conidia, indicating that these cells also play a role in defending against fungal infections [10].

PDT is closely related to the activation of macrophages, and research has shown that after ALA-PDT in a mouse model of Leishmania infection, markers related to the killing of intracellular parasites by activated macrophages are expressed at higher levels, the ratio of iNOS/arginase increases, indicating that PDT also affects macrophage polarization and enhances anti-infection effects [11]. PDT mediates immune response by regulating the interaction between immune cells, and *in vitro* studies have found that fibroblasts respond early to PDT and regulate the NF-κB pathway, which is activated to affect the secretion of cytokines by macrophages, increasing their production of pro-inflammatory and chemotactic factors [12]. PDT also can lead to immunogenic cell death of cells, resulting in damage-associated molecular patterns (DAMPs, including adenosine triphosphate (ATP), calreticulin (CRT), high-mobility group Box 1 (HMGB1), heat shock proteins (HSPs) 70 and 90, etc.) [13]. The combination of DAMPs and pathogen-associated molecular patterns‌‌ of pathogenic microorganisms with pattern recognition receptors (PRRs) of macrophages can lead to subsequent immune effects. HMGB1 plays an important role in chronic inflammation [14], and CBM is a disease with a chronic course. It is necessary to explore the role of HMGB1 in the pathogenesis of CBM. Additionally, research indicates that extracellular HMGB1 functions to trigger the innate immune system, inducing macrophages to undergo M1 activation to secrete pro-inflammatory cytokines and foster inflammatory reactions [14,15]. Therefore, the impact of HMGB1 on macrophages in the treatment of fungal infections with PDT remains to be further explored.

Therefore, this study will establish a mice footpad infection model, analyze the regulation and biological function of PDT on macrophages from the point of view of single-cell sequencing, and further investigate whether PDT affects the macrophage immune killing effect on *F. monophora* by inducing the release of HMGB1.

## 2 Materials and methods

### 2.1 Strains

The *Fonsecaea monophora* strain No. 212 was isolated from a patient with CBM examined in the Department of Dermatology and Venereology of our hospital in 2013. It has run ITS analyses (GenBank accession number: JN629041). The fungal strain was grown on Potato-dextrose agar medium, enriched with 40 mg/mL gentamicin sulfate injection, at a controlled temperature of 26°C for a duration of 14 days. Subsequently, the fungal cultures were meticulously washed and scraped from the medium surface, utilizing 0.9% NaCl for the purpose. The suspensions containing *F. monophora* cells, inclusive of conidia and yeast-like forms, were then adjusted to attain a concentration of 1 × 10^7^ CFU/mL, as determined through the Neubauer chamber method. Notably, the experimental protocol adhered to was duly approved by the Medical Ethics Committee of the third Affiliated Hospital of Southern Medical University.

### 2.2 Mouse model construction

A total of 15 female C57BL/6 (C57) mice aged 6–8 weeks were supplied by the Zhuhai Baishitong Biotechnology Co., Ltd. They were randomly assigned into five groups (three mice per group): healthy control group (uninfected), model group (infected without treatment), only light group (only red light), only ALA group (only ALA), and ALA-PDT group (ALA with red light).

The mice were anesthetized through intraperitoneal injection with a mixture of Xylazine Hydrochloride Injection, Zoletil 50 Injection, and 0.9% NaCl in a ratio of 1:1:18. In the healthy control group, mice were administered a volume of 0.1 mL of 0.9% NaCl to maintain their physiological health status as healthy controls. All mice belonging to the other four infectious groups underwent subcutaneous injection of 0.1 mL of *F. monophora* suspension into the footpad. Treatments were performed on the only ALA group, the only light group, and the ALA-PDT group at 7 days post-infection. All mice in each group were killed 6 h post-treatment. In this study, the animals were maintained under a controlled environment of a 12 h light/dark cycle, with unlimited access to food and water to ensure their welfare and consistency of experimental conditions.

### 2.3 Treatment

A vial containing 118 mg of ALA (Shanghai Fudan-Zhangjiang Bio-Pharmaceutical Co. Ltd, China) was dissolved in 0.4 mL of moisture gel, resulting in a solution with a concentration of 1.76 mol/L, equivalent to 25% ALA. Subsequently, medical cotton balls were soaked in this 25% ALA solution and applied as a topical dressing on the infected footpad skin. Subsequently, the skin was shielded from ambient light by a black plastic strip for a duration of 30 min. Ultimately, the skin area coated with ALA was exposed to a 635 nm red light emitting diode (LED) light source (Wuhan Yage Optic and Electronic Technique Co., Ltd, China). The irradiation was conducted with a light dose of 100 J/cm^2^ and an intensity of 331 mW/cm^2^, lasting for a period of 5 min. In the only ALA group, the mice were administered 25% ALA and shielded from light exposure for 30 min, without subsequent red LED irradiation. Additionally, the mice in the only light group underwent direct exposure to red LED light, adhering to identical treatment parameters as the ALA-PDT group.

### 2.4 Histopathology and mycology examination

Skin biopsies were collected from the footpads of mice belonging to each group, with specimens from the healthy group serving as controls. All biopsies were preserved using 10% formalin and subsequently embedded in paraffin for histological analysis. Thin sections, ranging from 3 to 5 μm in thickness, were stained with hematoxylin-eosin (HE) and carefully examined under a microscope. Subsequently, representative HE-stained images depicting inflammatory cells at the same time points within each group were carefully selected. In addition, subcutaneous tissue samples of infected footpads were collected for fungal culture and small culture examination. Samples of small culture examination are examined carefully under a microscope to detect fungal hyphae and conidia.

### 2.5 Single-cell mRNA sequencing

#### 2.5.1 Cell preparation

Skin biopsies from the footpads of the healthy control group, model group, and ALA-PDT group were collected and processed independently (*n* = 15 in total; each group three samples) as above. Upon harvesting, the tissues were rinsed with ice-cold RPMI1640 (Gibco, USA) and subsequently dissociated into a single-cell suspension using the Multi tissue Dissociation Kit 2 (Miltenyi Biotec, Germany), adhering strictly to the manufacturer’s guidelines. Before library preparation, cell suspensions were thawed, washed (phosphate buffer saline-bovine serum albumin 0.04%), filtered, and the cell viability was assessed; a viability cut-off of >70% was set for each sample to proceed.

#### 2.5.2 Single-cell RNA-seq library construction and sequencing

Single-cell RNA-Seq libraries were prepared using the SeekOne^®^ DD kit. Cells were mixed with reverse transcription reagent and added to Chip S3. Barcoded Hydrogel Beads and partitioning oil were dispensed into Chip S3 wells. Reverse transcription was performed at 42°C for 90 min and inactivated at 85°C for 5 min. cDNA was purified, amplified in polymerase chain reaction (PCR), cleaned, fragmented, repaired, A-tailed, and ligated to a sequencing adaptor. Indexed PCR was then performed to amplify the DNA representing the 3’ polyA part of expressing genes, containing cell barcode and unique molecular index. Libraries were cleaned with solid phase reversible immobilization beads, quantified by qPCR, and sequenced on Illumina NovaSeq 6000 or DNBSEQ-T7 with PE150 read length.

#### 2.5.3 Sequencing data quality control

Fastp (v0.20.1) trimmed primer sequences, low-quality bases, and collected statistics from raw reads. Key parameters: (1) Dropped bases in 1 bp sliding window if mean quality <3, moving from tail to front. Trimmed trailing N bases. (2) Detected auto adapter for PE data. (3) Discarded trimmed reads <60 bp. Cleaned reads used in subsequent steps.

#### 2.5.4 Processing the single-cell RNA sequencing data

Cell barcodes and UMI sequences were extracted based on localization patterns and corrected with a whitelist. Reads were mapped to reference genomes using STAR 2.5.1b. Reads with barcode and UMI info were assigned to transcriptome using featureCounts of Subread 1.6.4. Parameters of featureCounts varied with chemistries and regions. A raw UMI count matrix based on barcodes and transcripts was generated. A cell-calling algorithm filtered the matrix to obtain filtered_feature_bc_matrix. The algorithm identified cells based on total UMI counts and RNA profiles of each barcode.

#### 2.5.5 Clustering and visualization

Clustering and visualization were completed using Seurat with the following steps:

(1) Data normalization using LogNormalize, a global-scaling method.
(2) Detection of 2,000 highly variable features per dataset with FindVariableFeatures.
(3) Linear transformation (“scaling”) as a pre-processing step.
(4) Dimensional reduction using PCA on the scaled data, selecting the first 30 principal components.
(5) Clustering cells with a graph-based approach.
(6) Visualization and exploration using tSNE, a nonlinear dimensional reduction technique.
(7) Identification of cluster markers with FindAllMarkers, displaying the top nine markers.

#### 2.5.6 Gene ontology (GO) and Kyoto encyclopedia of genes and genomes (KEGG) enrichment analysis of marker genes

GO enrichment analysis of marker genes was conducted using the ClusterProfiler R package, with gene length bias correction. GO terms with corrected *P* < 0.05 were considered enriched.

KEGG is a database for understanding high-level functions of biological systems from molecular-level information. We used ClusterProfiler to test the enrichment of marker genes in KEGG pathways with corrected *P* < 0.05.

### 2.6 Quantitative real time-PCR (qPCR)

Extraction of isolated total RNA from tissues was achieved by Trizol Reagent (Gibco, USA). Expression levels of genes (HMGB1) were analyzed by qPCR. The purity and concentration of RNA were determined using a MicroDrop spectrophotometer (NanoDrop 2000, USA). Next reverse transcription of cDNA and quantitative real-time PCR reactions were performed using All-in-One^TM^ qPCR Mix (GeneCopoeia, USA). The cycling program was as follows: 95°C for 10 s, followed by 40 cycles of 95°C for 10 s, and 60°C for 30 s. The melting curves exhibited a temperature range varying from 60 to 95°C. All primers were synthesized in WooTech Biotech (Table 1). All expression data were normalized to β-actin. The relative expression of genes was calculated using the power formula: 2^−ΔΔCt^.

**Table 1:** Primer sequences used to amplify genes

Primer	Sequence (5′−3′)
HMGB1	Fwd-GGCTGCTTGTCATCTGCTG
	Rev-GGCGAGCATCCTGGCTTATC
β-actin	Fwd-CCACCATGTACCCAGGCATT
	Rev-CAGCTCAGTAACAGTCCGCC

### 2.7 Western blot assay

Total proteins from footpad skin tissues were lysed in chilled radio-immunoprecipitation assay buffer (Ubio, China) with inhibitors. Protein concentrations were measured using a Pierce BCA kit (Ubio, China). Equal proteins were loaded onto sodium dodecyl sulfate-polyacrylamide gel electrophoresis gels and transferred to polyvinylidene fluoride membranes (Millipore, USA). Membranes were blocked in 5% milk for 1 h and incubated with primary antibodies overnight at 4°C. Used antibodies: anti-HMGB1 (1:700, Biodragon, China). After washing with tris-buffered saline with Tween-20 buffer three times for 10 min each time, membranes were incubated with horseradish peroxidase-conjugated goat anti-mouse immunoglobulin G (1:10,000, UBio, China) for 1 h. Proteins were visualized using an electrochemiluminescence Western Blotting Substrate (Ubio, China) and AlphaView gel imaging system.

### 2.8 Enzyme-linked immunosorbent assay

Cytokine levels, specifically HMGB1, IFN-γ, IL-4, IL-10, and IL-12, were measured in mouse serum utilizing a commercially available ELISA kit in accordance with the manufacturer’s guidelines. The optical density of each ELISA sample was precisely assessed with a microplate reader set at a wavelength of 450 nm. The concentrations of cytokines were then determined through the application of a standard curve.

### 2.9 Statistical analysis

The statistical significance of the data was evaluated using the GraphPad Prism software. The results are expressed as mean values ± SD. Analysis of Variance (ANOVA) was used to determine the significance between multiple groups. Statistical significance was defined as *P* < 0.05.


**Ethics statement:** This study was duly approved by the Experimental Animal Ethics Committee of Guangdong Huawei Testing Co., Ltd, with approval number 202207002. Strict adherence to the established standard guidelines for the use and care of laboratory animals was observed throughout the experimental procedures.

## 3 Results

### 3.1 Successful establishment of the infection model in murine footpad has been achieved

**Figure 1:**
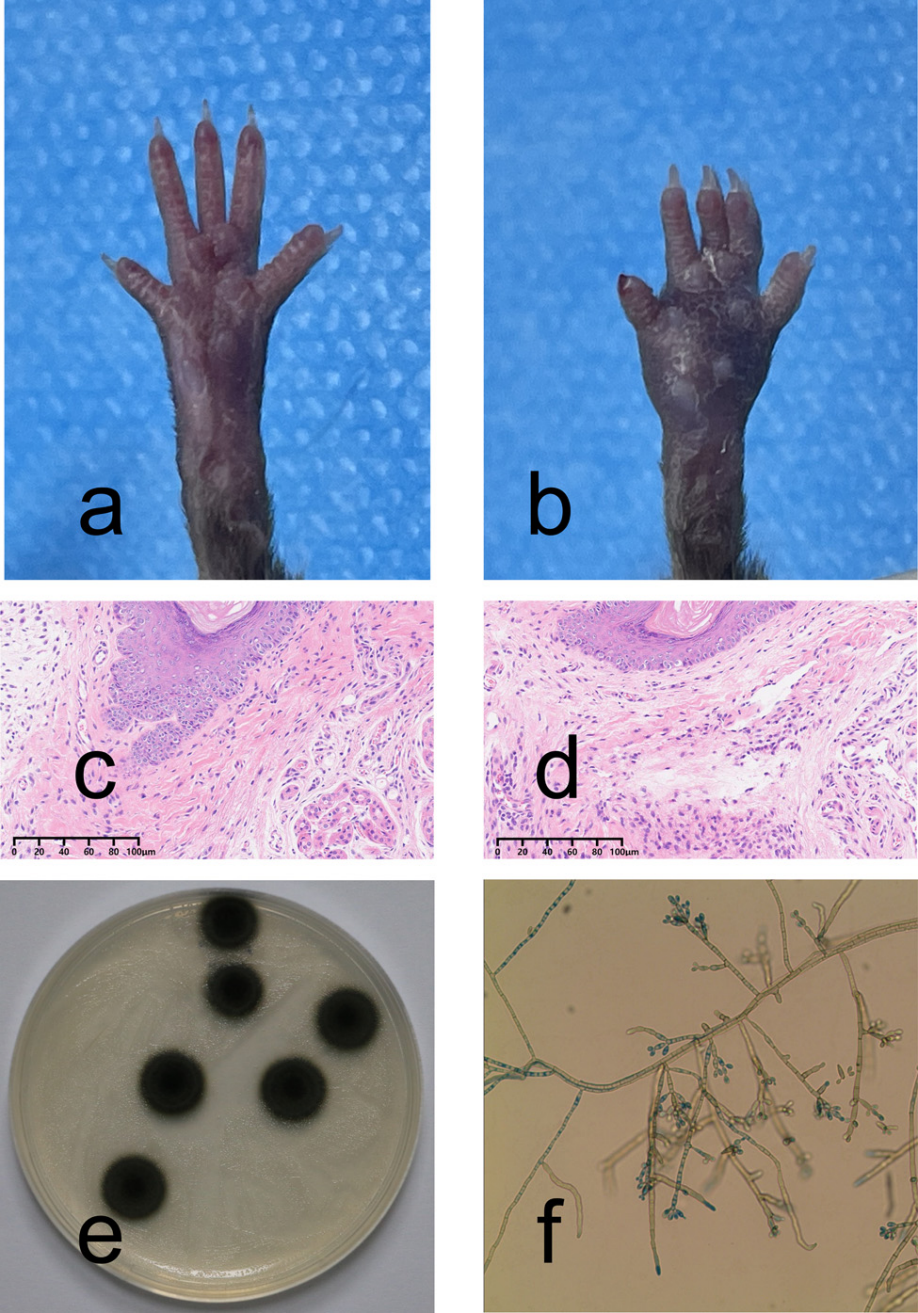
C57BL/6 murine footpad infection model with *F. monophora* and representative images of mycological and pathological examination. (a) Clinical manifestation of the footpads. (b) After inoculation with *F. monophora*, the infected foot pad showed an obvious inflammatory response, characterized by swelling and a small amount of desquamation. (c) Histopathological examination of the control group (HE ×200). (d) Inflammatory cells infiltration was observed after inoculation with *F. monophora*, by histopathological examination (HE ×200). (e) and (f) Fungal culture and micro-culture results.

The clinical manifestations of footpads are shown in Figure 1. After inoculation with *F. monophora*, the infected footpad showed an obvious inflammatory response, characterized by swelling and a small amount of desquamation (Figure 1b). Histopathological examination of footpads is shown in Figure 1c and d. Histopathological examination showed an increase in inflammatory cells (Figure 1d). Fungal culture results are shown in Figure 1e and f. Fungal culture reveals black colonies with gray short hairy mycelia on the surface (Figure 1e). In micro-culture, lactophenol cotton blue staining was used, and under the microscope, brown hyphae with septa and branching were visible, as well as conidiophores (Figure 1f).

### 3.2 Cell clustering analysis

**Figure 2:**
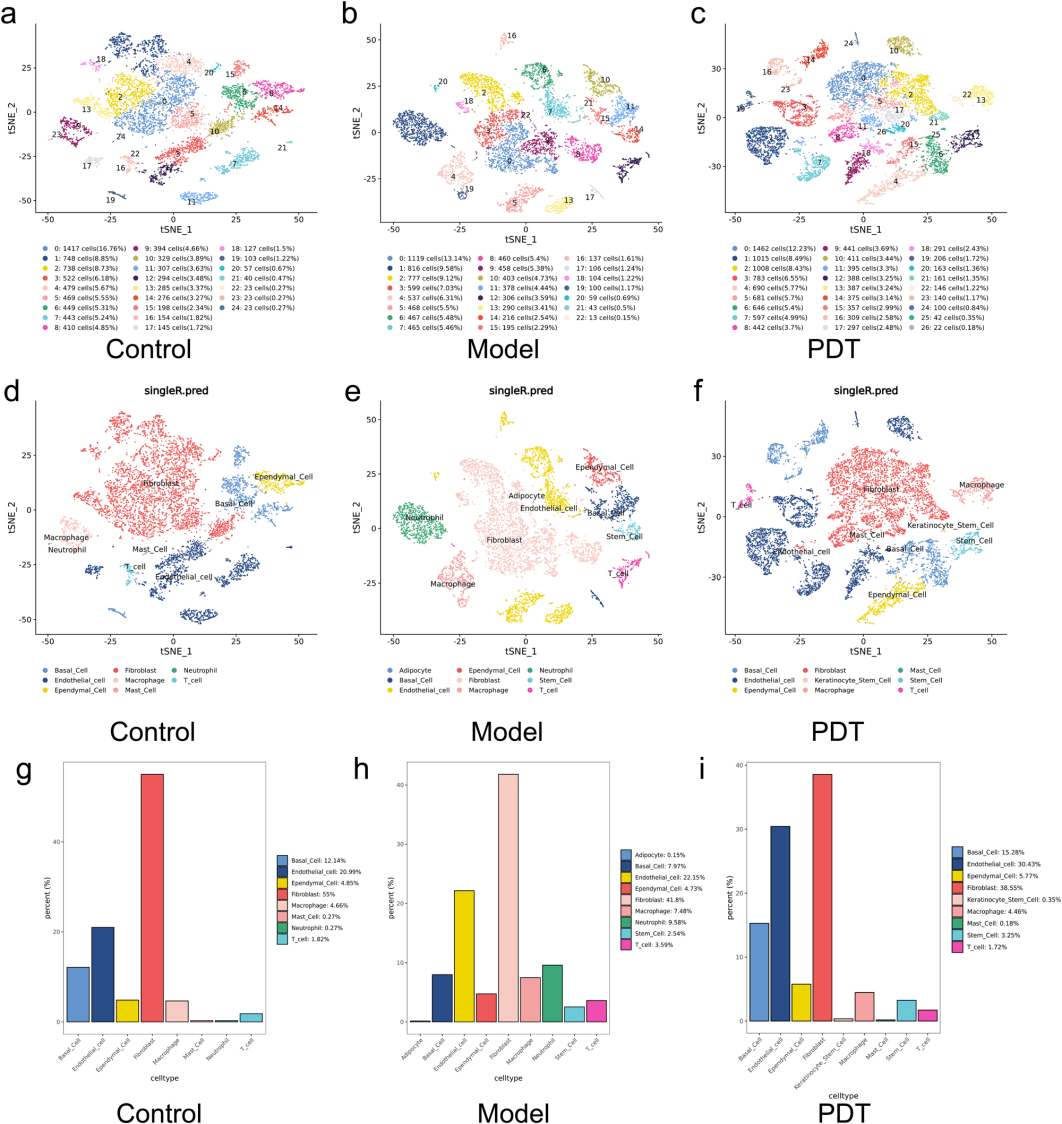
Cell clustering graph, corresponding cell annotation results, and plots of the percentage of cell types in each sample of the three groups. (a)–(c) Cell clustering plots for samples from the control, model, and ALA-PDT groups. (d)–(f) Cell annotation plots corresponding to cell clustering for samples from the control, model, and ALA-PDT groups. (g)–(i) The percentage of cell types in each sample from the control, model, and ALA-PDT groups.

The clustering results of the cell subpopulations in the three groups (healthy control group, model group, and ALA-PDT group) are shown in Figure 2a–f, with annotations for the different cell types. In combination with Figure 2g–i, which shows the proportion of different cell types, it can be seen that regardless of the grouping, the most abundant cell type is the fibroblasts, accounting for 72–87% of the total. This is related to the composition of the dermal cells in skin tissue. The proportion of immune cells varies among the three groups, with the model group having a higher proportion of monocytes, macrophages, T cells, and granulocytes than the other two groups, indicating that the inflammatory cells were activated after modeling. In the ALA-PDT treatment group, no monocytes or granulocytes were found, and the proportion of macrophages and T cells decreased to normal group levels, indicating that PDT can improve the inflammatory response.

### 3.3 Marker gene analysis

**Figure 3:**
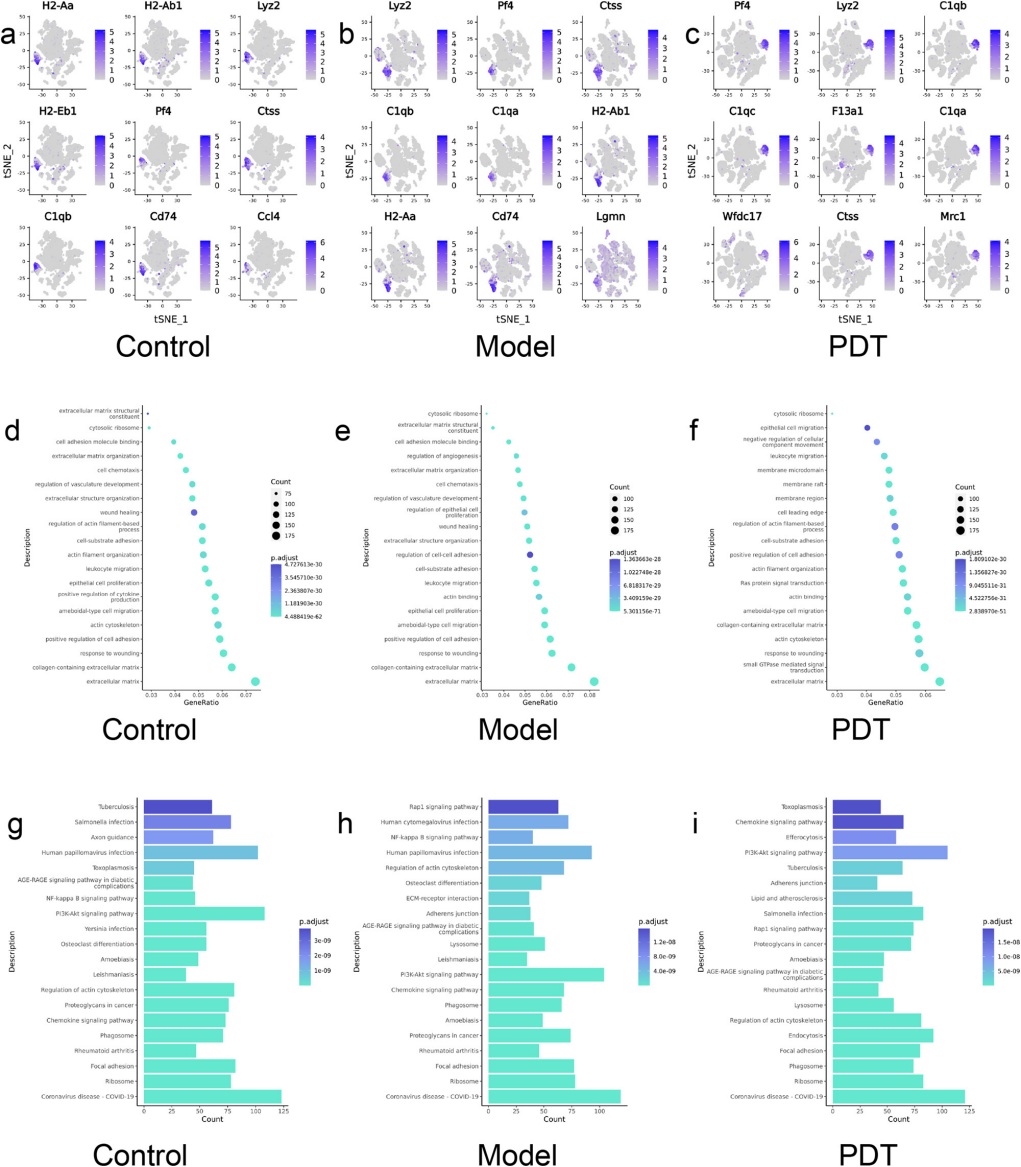
Differential gene expression, GO function enrichment, and KEGG pathway enrichment in macrophage cluster in three groups of samples. (a)–(c) Differential gene expression in macrophage cluster in three groups of samples; (d)–(f) Scatterplot of GO function enrichment for three groups of samples; (g)–(i) Histogram of KEGG pathway enrichment in three groups of samples.

**Figure 4:**
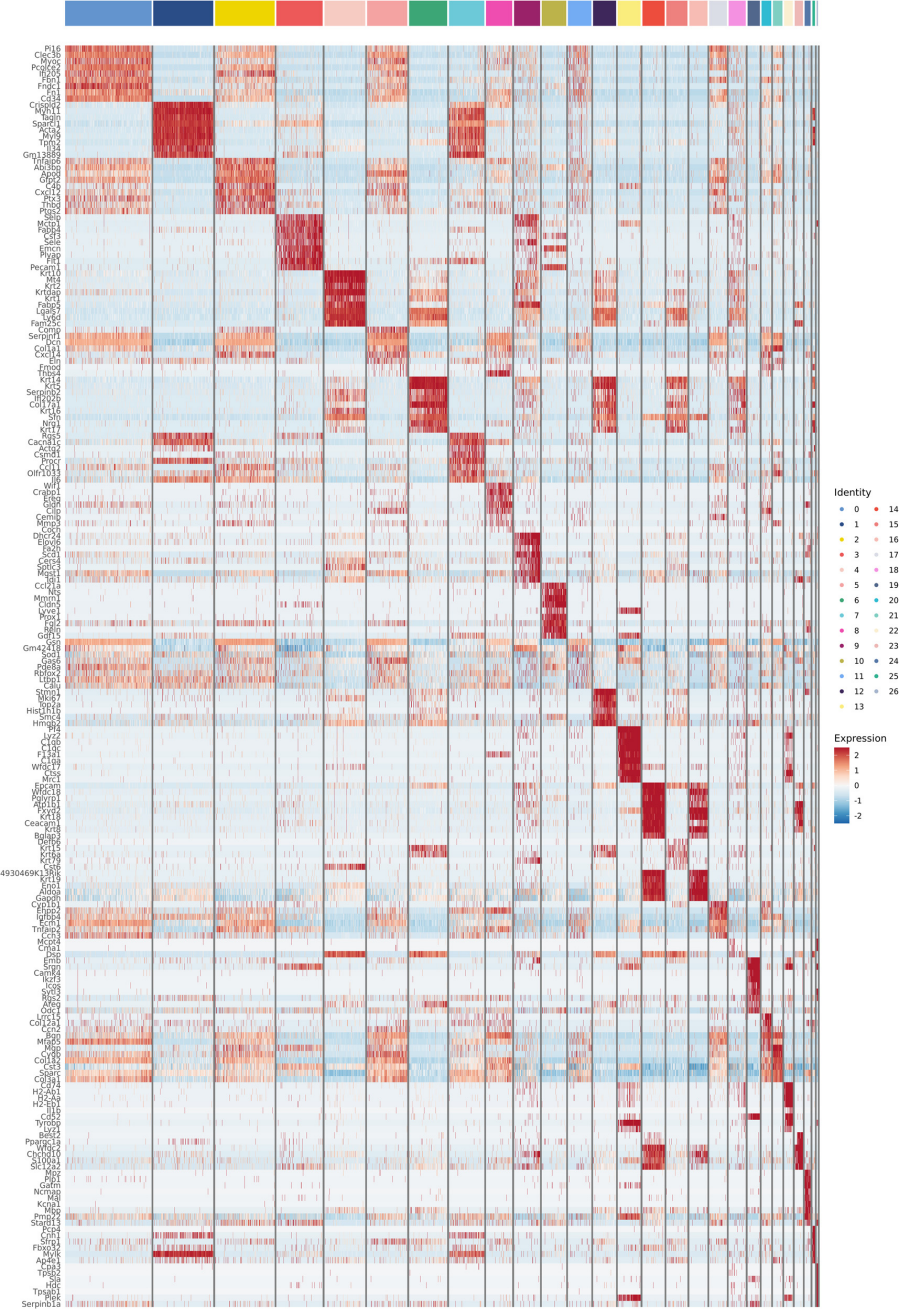
Heatmap of overall clustering of ALA-PDT group marker genes. Each row represents a gene and each column represents a cell type, where the yellow squares at serial number 13 represent macrophage clusters. Red color in the heatmap suggests high expression and the blue color suggests low expression.

By calculating the differential genes between subsets and other subsets, specific highly expressed genes in subsets can be obtained. Figure 3a–c shows the top nine differential genes highly expressed by macrophages in three groups of specimens, and by comparing the marker genes of macrophages in different treatment groups, it can be inferred that the cell function changes caused by different stimuli or treatments. Combining Figure 4 with the clustering heatmap, it can be seen that the model group has a higher expression of complement C1qa, CD74 molecule, Lgmn, etc., compared to the normal group; after PDT treatment in the infection model, the genes C1qc, F13a1, Ctss, Mrc1, etc., were upregulated.

Figure 3d–f shows the results of the GO data of each sample based on its own molecular function, biological process, and cell composition, and selects the most significant 30 entries. It can be seen that compared with the model group, epithelial proliferation, and cell chemotaxis were enhanced, while the ALA-PDT group upregulated actin filament remodeling, actin filament-based activity, Ras protein signaling, and enzyme activation compared with the model group.

The KEGG enrichment results for the three groups of samples are shown in Figure 3g–i and the expression patterns of macrophage-related functional pathways between different treatment groups are compared. The model group is enriched in pathways mainly related to anti-infection signaling pathways activated by viruses, protozoa, and other pathogens, which is consistent with the fungal infection model of the skin. The ALA-PDT group showed significant enhancement of macrophages in activities such as phagocytosis, endocytosis, chemokine-related signaling pathways, regulation of lysosomes, and phagosome function compared to the model group.

### 3.4 ALA-PDT leads to the decreased expression of HMGB1

**Figure 5:**
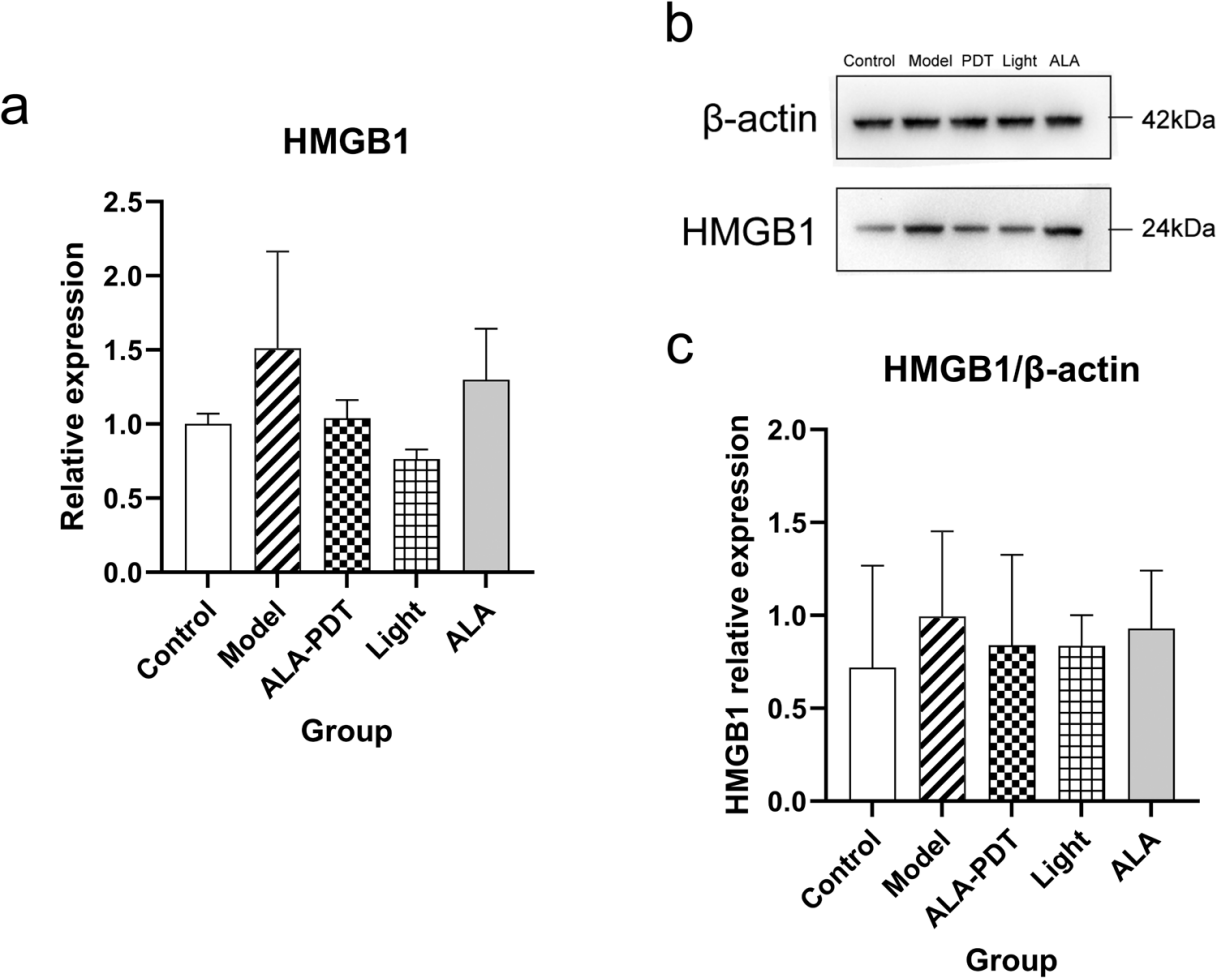
The expression of the HMGB1 gene used by qRT-PCR and the expression of the HMGB1 protein used by western blotting analysis. (a) The RNA expressions of HMGB1 gene in footpad skin used by qRT-PCR; (b) and (c) Western blot shows the protein expression and relative grey value of HMGB1 in five groups of samples.

At the gene level, we selected the HMGB1 gene (one of the ICD-related genes), to analyze the expression by qRT-PCR in Figure 5a. The results showed that, compared with the model group, the expression level of the HMGB1 gene in the ALA-PDT group was downregulated.

At the protein level, HMGB1 proteins were quantified by verification with Western blot analysis in Figure 5b and c. Compared with healthy mice, the expression level of HMGB1 protein in the model group, the only light group, the only ALA group, and the ALA-PDT group increased. In addition, the expression level of HMGB1 protein in the other three groups was lower than that in the model group. However, there was no statistical difference between the five groups.

### 3.5 Measurement of serum cytokines

**Figure 6:**
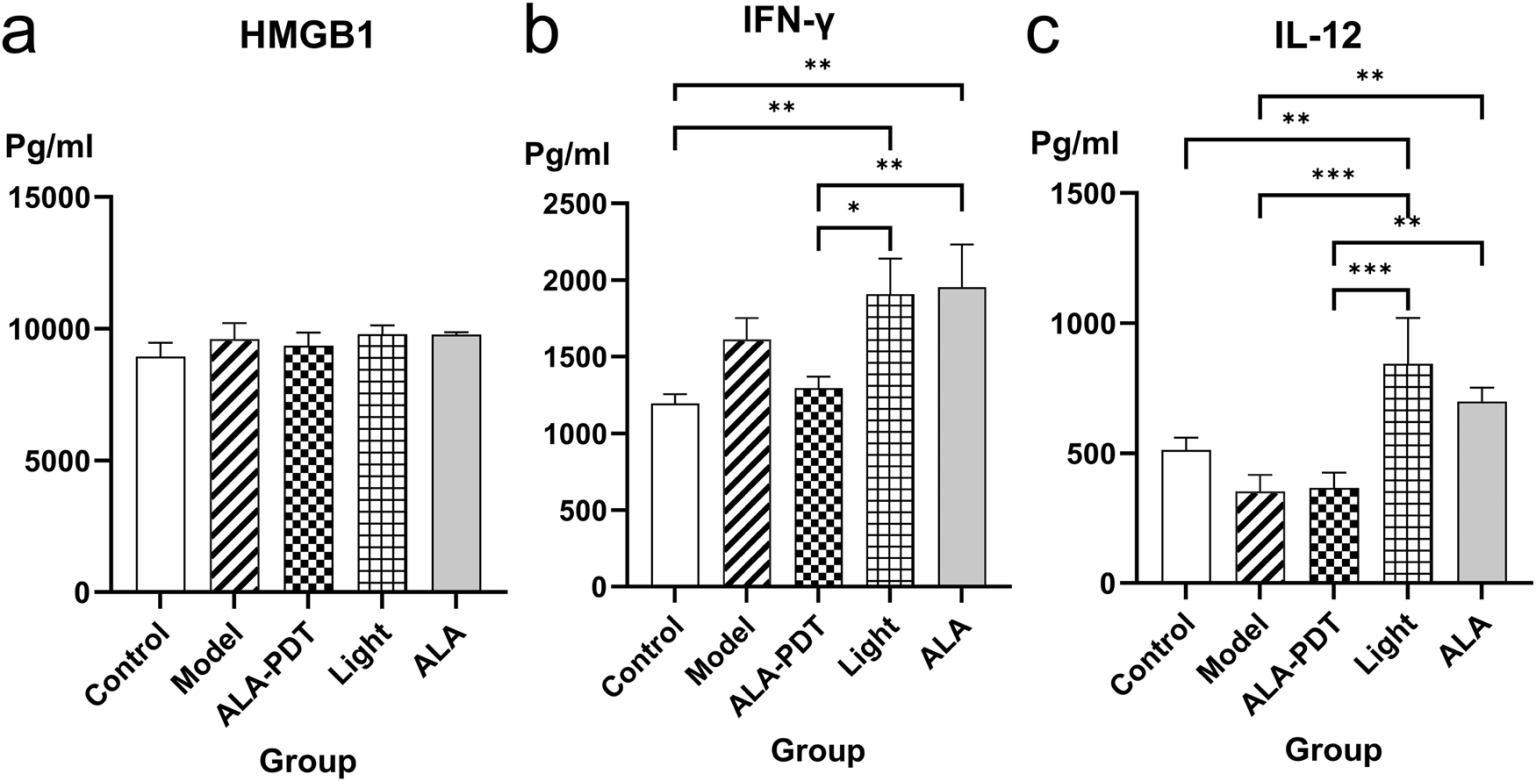
The expression of serum cytokines (a) HMGB1, (b) IFN-γ, and (c) IL-12.

Utilizing the technique of ELISA, we identified five cytokines that represent the immune response mounted by the host against the *F. monophora*. We compared the expression levels of cytokines among the five groups. The results showed that compared with the control group, the expression levels of HMGB1 and IFN-γ in the other four groups were increased in Figure 6a and b. The expression levels of IL-12 were decreased in the model group and the ALA-PDT group in Figure 6c. We also compared the levels of cytokines in the model group and the ALA-PDT group. PDT decreased the level of pro-inflammatory cytokine IFN-γ but increased the level of IL-12. Meanwhile, PDT increased the level of anti-inflammatory cytokines IL-4 and IL-10 (the results of IL-4 and IL-10 will be published in another article). In addition, compared with the model group and the ALA-PDT group, the expression levels of IFN-γ and IL-12 in the only light group and the only ALA group increased.

## 4 Discussion

Based on the results of cell clustering analysis, it can be seen that after skin infection with fungi, there is a significant increase in the proliferation and proportion of inflammatory cells such as monocytes/macrophages and neutrophils. After PDT treatment, the proportion of macrophages decreases and returns to normal levels, while the number of neutrophils significantly decreases. This indicates that PDT can reduce the proliferation and aggregation of inflammatory cells and alleviate inflammatory reactions. Additionally, from the analysis of marker genes, it can be seen that there are differences in gene expression between the three groups of samples, with macrophages in the model group expressing CD74, which is a membrane-bound subunit of major histocompatibility complex II-related invariant chains and primarily marks B lymphocytes and dendritic cells [16]. The interaction between CD74 and macrophage migration inhibitory factor cytokines can activate multiple pathways that are crucial for cell survival, differentiation, and proliferation. The genes that are highly expressed by macrophages in the ALA-PDT group are mainly involved in antigen recognition, presentation, and other processes that play important roles in antigen presentation. Mrc1 is a mannose receptor C1, a member of the C-type lectin superfamily, which is expressed on the surface of macrophages and recognizes and binds specific sugar molecules through its extracellular region. It plays a role in recognizing pathogens, antigen presentation, and maintaining internal environment stability [17]. Ctss encodes pro-cysteine protease, which is a lysosomal cysteine protease involved in degrading antigenic proteins and antigen presentation [18]. Lyz2 is a lysozyme-related gene that is closely related to the mononuclear-macrophage system and enhances immune activity [19]. Therefore, it can be seen that ALA-PDT mainly enhances the expression of genes related to antigen recognition, presentation, internalization, digestion, and other functions in macrophages, thereby improving the immune killing of pathogens in the body.

The results of GO and KEGG enrichment for PDT groups indicate that both are associated with the biological functions of macrophage phagocytosis and autophagy activity, as well as functions such as actin filament remodeling and regulation of the actin cytoskeleton. KEGG enrichment also includes pathways related to phagocytosis, intracellular degradation, autophagosomes, and lysosomes. In combination with differential marker gene analysis, PDT may regulate the expression of PRRs on macrophages, activate macrophages, and enhance their antigen recognition and processing ability. Some studies have shown that PDT can lead to immunogenic cell death, and DAMPs (includes ATP, CRT, HMGB1, HSPs 70 and 90) which are exposed or released during the process of cell death can combine with PRRs of immune cells, promoting the recruitment and maturation of antigen-presenting cells [13,20].

HMGB1 is considered a structural chromatin-binding factor that is involved in the maintenance of nucleosome structure and gene transcription, with multiple biological functions such as maintaining chromatin stability and participating in cellular DNA rearrangement [21,22,23,24]. Extracellular HMGB1 also regulates various biological processes such as cell differentiation, cell migration and metastasis, apoptosis, and inflammatory responses [25]. Studies have shown that extracellular HMGB1 can activate the innate immune system as a DAMP, recruiting inflammatory cells and smooth muscle cells, stimulating macrophages and endothelial cells to produce pro-inflammatory cytokines and promote inflammatory responses [13]. Additionally, as an immune molecule, extracellular HMGB1 can trigger inflammatory responses in immune cells and endothelial cells. At present, the identified origins of extracellular HMGB1 primarily adhere to two distinct mechanisms: active secretion and passive release. Active secretion: Upon stimulation by LPS, TNF-α, IFN-γ, IL-1, and similar factors, macrophages, monocytes, pituitary cells, epithelial cells, and other cell types actively secrete HMGB1 extracellularly through an unconventional vesicle-mediated process. Passive release: HMGB1 is released following cellular injury or necrosis. However, in addition, immune cells and endothelial cells activated by HMGB1 can secrete HMGB1, forming a positive feedback loop. Therefore, HMGB1 can maintain a long-term inflammatory state under various stimulating conditions. Additionally, HMGB1 can induce M1 polarization in macrophages [15]. In this study, compared with the control group, the gene and protein expression level of HMGB1 was upregulated in both the model group and the ALA-PDT group, and the expression level of HMGB1 in the model group was slightly higher than that in the PDT group. It shows that in the *F. monophora*-infected mice model, ALA-PDT can down-regulate the expression of HMGB1. The serum cytokines suggest that the expression level of HMGB1 in the model group and the ALA-PDT group is higher than that in the control group, but the difference between the two groups is not significant. Studies have already proven that M2 macrophages have a stronger ability to phagocytose cells *in vitro* than M1 macrophages and they are more inclined to phagocytose early apoptotic cells rather than late apoptotic cells and necrotic cells [15,26]. Therefore, ALA-PDT may reduce the inflammatory response by reducing the release of HMGB1, and lead to M2 polarization in macrophages to protect the host from damage caused by excessive inflammatory reactions.

The effect of ALA-PDT on the immune microenvironment was analyzed through the analysis of mice serum cytokines. After the mice’s footpads were infected with *F. monophora*, the expression of IFN-γ increased while the expression of IL-12 decreased, which was similar to previous reports. A previous study on *F. monophora* suggested that *F. monophora* could evade the antifungal response by binding to C-type lectin receptor mincle and inhibit IL-12 [27]. Another report on the infection of *F. pedrosoi-spores* in mice showed that IFN-γ increases in the early stage of infection [28]. After PDT treatment, the expression of inflammatory cytokines IFN-γ was downregulated, while the level of anti-inflammatory cytokine (IL-4, IL-10) was upregulated. This observation indicates that ALA-PDT induced an increase in anti-inflammatory cytokines while suppressing the levels of pro-inflammatory cytokines in the serum of the mice infection model. As a result, PDT effectively modulated the inflammatory state in infected mice, shifting it from a pro-inflammatory to an anti-inflammatory condition. The decrease in anti-inflammatory factors is similar to previous reports on PDT [29]. Furthermore, IFN-γ plays a pivotal role in orchestrating the polarization of macrophages toward the M1 phenotype, whereas IL-4 and IL-10 trigger the transition of macrophages toward the M2 phenotypic configuration. In this study, ALA-PDT exhibits the potential to induce M2 polarization in macrophages, thereby safeguarding the host from detrimental effects resulting from excessive inflammatory responses and fostering tissue repair processes. Moreover, compared with the model group and the ALA-PDT group, the expression levels of IFN-γ and IL-12 in the only light group were increased. This result is similar to the previous research conclusion about the treatment of red light-emitting diode in the experimental model of acute lung injury induced by sepsis. LED treatment can enhance the concentration of IFN-γ, which may help to regulate immune response [30]. There is a positive feedback loop between IFN-γ and IL-12: the secretion of IFN-γ induces the production of IL-12, and IL-12 further induces the secretion of IFN-γ [31]. The intricate mechanisms that underlie the immune microenvironmental modulation induced by PDT demand deeper and more comprehensive exploration. Understanding these mechanisms is essential in order to gain a comprehensive appreciation of how PDT influences and shapes the immunological milieu.

The limitation of this study lies in the fact that it did not delve deeper into the potential signaling pathways that might be involved in the mechanism of ALA-PDT against *F. monophora*. Moreover, the brevity of the study duration also constitutes a restriction, leaving certain aspects unexplored. At the same time, this experiment only detected the changes in HMGB1 level after 6 h of treatment, without monitoring its long-term changes, and did not fully explore the changes in other DAMPs. The subsequent antigen presentation function caused by macrophages after PDT has not been further explored. Additionally, this study only discusses the effects of ALA-PDT on macrophages and lacks further exploration of the potential impacts on other types of immune cells (such as T cells and dendritic cells), which remains to be explored.

## 5 Conclusion

To investigate cell types and functions affected by ALA-PDT in a mouse footpad *F. monophora* infection model, single-cell transcriptomic sequencing was performed on skin samples from mice in three conditions: normal, infected, and treated. Bioinformatics analysis was conducted. After infection, inflammatory cells such as monocytes-macrophages and granulocytes increased significantly. However, following PDT treatment, the number and proportion of macrophages decreased, indicating anti-inflammatory effects. Marker gene analysis revealed that PDT enhanced macrophage antigen recognition, presentation, and degradation. Combined with GO and KEGG results, PDT strengthened actin-based functions and upregulated anti-infection pathways including phagocytosis, autophagy, and cell clearance. We initially analyzed the potential mechanism of how ALA-PDT exerts its influence on macrophages within the infected model, thereby establishing a solid groundwork for subsequent investigations into the intricate mechanisms underlying PDT for CBM. This exploration serves as a pivotal step in advancing our understanding of the therapeutic modalities for this challenging condition.
